# Influence of age on respiratory modulation of muscle sympathetic nerve activity, blood pressure and baroreflex function in humans

**DOI:** 10.1113/EP085071

**Published:** 2015-08-18

**Authors:** Alena Shantsila, David B. McIntyre, Gregory Y. H. Lip, Paul J. Fadel, Julian F. R. Paton, Anthony E. Pickering, James P. Fisher

**Affiliations:** ^1^ School of Sport, Exercise & Rehabilitation Sciences, College of Life and Environmental SciencesUniversity of BirminghamBirminghamUK; ^2^ University of Birmingham Centre of Cardiovascular Sciences, City HospitalBirminghamUK; ^3^ Medical Pharmacology & Physiology, Dalton Cardiovascular Research CenterUniversity of MissouriMOUSA; ^4^ School of Physiology & Pharmacology, Bristol CardioVascularUniversity of BristolBristolUK

## Abstract

**New Findings:**

- **What is the central question of this study?**
  Does ageing influence the respiratory‐related bursting of muscle sympathetic nerve activity (MSNA) and the association between the rhythmic fluctuations in MSNA and blood pressure (Traube–Hering waves) that occur with respiration?
- **What is the main finding and its importance?**
  Despite the age‐related elevation in MSNA, the cyclical inhibition of MSNA during respiration is similar between young and older individuals. Furthermore, central respiratory–sympathetic coupling plays a role in the generation of Traube–Hering waves in both young and older humans.

Healthy ageing and alterations in respiratory–sympathetic coupling have been independently linked with heightened sympathetic neural vasoconstrictor activity. We investigated how age influences the respiratory‐related modulation of muscle sympathetic nerve activity (MSNA) and the association between the rhythmic fluctuations in MSNA and blood pressure that occur with respiration (Traube–Hering waves; THW). Ten young (22 ± 2 years; mean ± SD) and 10 older healthy men (58 ± 6 years) were studied while resting supine and breathing spontaneously. MSNA, blood pressure and respiration were recorded simultaneously. Resting values were ascertained and respiratory cycle‐triggered averaging of MSNA and blood pressure measurements performed. The MSNA burst incidence was higher in older individuals [22.7 ± 9.2 *versus* 42.2 ± 13.7 bursts (100 heart beats)^−1^, *P* < 0.05], and was reduced to a similar extent in the inspiratory to postinspiratory period in young and older subjects (by ∼25% compared with mid‐ to late expiration). A similar attenuation of MSNA burst frequency (in bursts per minute), amplitude and total activity (burst frequency × mean burst amplitude) was also observed in the inspiratory to postinspiratory period in both groups. A significant positive correlation between respiratory‐related MSNA and the magnitude of Traube–Hering waves was observed in all young (100%) and most older subjects (80%). These data suggest that the strength of the cyclical inhibition of MSNA during respiration is similar between young and older individuals; thus, alterations in respiratory–sympathetic coupling appear not to contribute to the age‐related elevation in MSNA. Furthermore, central respiratory–sympathetic coupling plays a role in the generation of Traube–Hering waves in both healthy young and older humans.

## Introduction

Healthy ageing is associated with elevated plasma catecholamine concentrations, increased noradrenaline spillover from the heart, brain and kidneys and greater sympathetic neural activity directed to the skeletal muscle vasculature (muscle sympathetic nerve activity, MSNA; Sundlöf & Wallin, 1978; Seals & Esler, 2000). Such heightened sympathetic nerve activity has been linked to structural and functional abnormalities of the peripheral vasculature (e.g. increased arterial stiffness, impaired endothelial function) in several chronic disease states (Grassi *et al*. 1995; Failla *et al*. 1999) and in elderly individuals (Thijssen *et al*. 2006). Alongside increases in tonic MSNA, α‐adrenergic receptor sensitivity and vascular responsiveness are reportedly diminished with increased age (Dinenno *et al*. 2005; Vianna *et al*. 2012). The mechanistic basis for the age‐related elevation in sympathetic neural firing remains unclear.

It has been known since the earliest direct recordings that sympathetic nerve activity shows respiratory modulation (Adrian *et al*. 1932). This is generated in large part by central neural circuits (Häbler *et al*. 1994), upon which is superimposed modulatory feedback signals from cardiorespiratory afferents that include lung‐stretch receptors, baroreceptors and central and peripheral chemoreceptors (Dempsey *et al*. 2002). During normal breathing in young healthy individuals, MSNA is inhibited during mid‐inspiration, reaching a nadir when lung volume is at its highest (peak inspiration) and peaking when lung volume is at its lowest (end expiration; Eckberg *et al*. 1985, 1988; Seals *et al*. 1990; St Croix *et al*. 1999; Dempsey *et al*. 2002). Patients with chronic heart failure show increased MSNA linked to an attenuation of the normal inspiratory sympathoinhibition (Goso *et al*. 2001). Given the increase in the tonic level of MSNA with age, we may predict a reduction in the inspiratory inhibition of MSNA; however, there is a paucity of information on the effect of ageing on respiratory–sympathetic coupling. Fatouleh & Macefield (2011) reported a similar pattern of respiratory modulation of MSNA in young (29 ± 2 years old) and middle‐aged groups (50 ± 3 years old) using cross‐correlation histograms constructed between sympathetic spikes and respiratory‐related chest excursions. However, the respiratory modulation of MSNA was not assessed in terms of sympathetic burst occurrence (i.e. incidence) or strength (i.e. amplitude; Malpas & Ninomiya, 1992; Malpas *et al*. 1996; Sverrisdottir *et al*. 2000; Kienbaum *et al*. 2001 *a*); thus, whether ageing affects the within‐breath modulation of these distinct parameters of sympathetic nerve activity is unclear.

The respiratory modulation of vasomotor sympathetic outflow causing phasic changes in arteriolar smooth muscle tone generates Traube–Hering arterial blood pressure (BP) waves (THWs). Importantly, Simms *et al*. (2009) demonstrated that spontaneously hypertensive rats exhibited augmented respiratory–sympathetic coupling and larger THWs that contributed more to vascular resistance than in normotensive Wistar–Kyoto control rats at all ages (Simms *et al*. 2009). However, the contribution of the sympathetic nervous system to the regulation of THWs in humans remains controversial, perhaps as a consequence of the multiple mechanisms implicated and the methodological approaches previously used to investigate this (e.g. complete pharmacological autonomic blockade, individuals with brain death; Conci *et al*. 2001; Zhang *et al*. 2002). In a recent preliminary investigation employing a novel time‐domain analysis, Towie *et al*. (2012) revealed a significant positive correlation between respiratory‐mediated changes in MSNA and the following THWs in a group of young individuals (age 21–30 years). It was suggested that this finding supported the hypothesis that THWs are partly a consequence of central respiratory–sympathetic coupling in humans (Towie *et al*. 2012). However, it is incompletely understood whether there is an alteration in respiratory coupling of MSNA with healthy human ageing; whether this could account for the increased MSNA seen with ageing; and if this sympathetic flux still gives rise to THWs.

The purpose of the present study was to determine the influence of age on respiratory modulation of MSNA and BP in humans. We tested the hypothesis that given the increase in MSNA during healthy ageing there would be diminished inspiratory inhibition of MSNA. Furthermore, we anticipated that the sympathetic contribution to respiration‐mediated fluctuations in BP (THWs) would also be attenuated in older individuals based on reported sympathetic end‐plate deficiencies (Esler *et al*. 1995; Dinenno *et al*. 2005).

## Methods

### Subjects

Ten young (mean ± SD, 22 ± 2 years) and 10 older men (58 ± 6 years) participated in the study, which was approved by the local ethical review committee and conducted in accordance with the *Declaration of Helsinki* (2000). All participants provided written informed consent before they took part in any experiments. Participants were healthy, with no significant medical history, and were not taking any prescription or over‐the‐counter medications. All subjects were asked to abstain from caffeine use for at least 12 h and from alcohol intake and strenuous physical activity for at least 24 h prior to the participation. All study measurements were made in a temperature‐controlled room (20–22°C).

### Experimental measurements

All measurements were obtained while subjects rested in a supine position and breathed spontaneously at a normal resting rate and depth. The following recordings were obtained simultaneously: (i) a lead II ECG; (ii) arterial BP from the middle finger using photo‐plethysmography (Finometer Pro; Finapres Medical Systems BV, Arnhem, The Netherlands); and (iii) respiratory‐related changes in thoracic circumference using a strain‐gauge pneumobelt placed securely around the upper abdomen (Pneumotrace; UFI, Morro Bay, CA, USA). Respiration was monitored in this way because it is unobtrusive and avoids participants having to breathe through a mouthpiece, which may alter breathing pattern (Han *et al*. 1997; Peng *et al*. 2002). In addition, recordings of postganglionic multiunit MSNA were obtained using standard microneurography techniques (unipolar tungsten microelectrodes). Briefly, a microelectrode was placed into the peroneal nerve at the fibular head and a reference electrode inserted subcutaneously at a site 2–3 cm distal. Prior to digitization, raw signals were amplified (×100,000), filtered (bandwidth 700–2000 Hz), rectified and integrated (time constant 100 ms) in order to obtain a mean voltage neurogram. An MSNA recording was considered acceptable if it displayed a pulse‐synchronous pattern of spontaneous bursts, had a signal‐to‐noise ratio of 3:1, was increased during an end‐expiratory breath‐hold or Valsalva manoeuvre and was unresponsive to an unexpected loud noise or skin stroking (Sundlöf & Wallin, 1977). After obtaining an MSNA signal, participants rested for at least 10 min to confirm recording stability, after which all study measures were obtained over an additional 10 min period.

### Data analyses

The raw signals underwent analog‐to‐digital conversion at 1 kHz (Powerlab and Chart v7; AD Instruments, Bella Vista, NSW, Australia) and were stored for offline analysis. Heart rate (HR) was calculated on a beat‐to‐beat basis from the ECG. Beat‐to‐beat systolic and diastolic BP were obtained from the arterial BP waveform, and mean arterial pressure (MAP) was obtained by integration of the arterial BP waveform over the entire cardiac cycle. Peak inspiration was defined as the highest point of the pneumobelt waveform, and respiratory rate was calculated from the inspiratory peaks. The MSNA bursts were identified using a custom‐written interactive scoring program (Spike2; Cambridge Electronic Design, Cambridge, UK). Sympathetic neurograms were shifted in time to account for conduction delays (1.35 ± 0.03 s) calculated according to subject height (Fagius & Wallin, 1980) as in previous studies investigating respiratory–sympathetic coupling (Eckberg *et al*. 1985; Seals *et al*. 1990, 1993). The neurogram baseline was established (designated zero), and the amplitude of the highest spontaneous burst was assigned a value of 100 arbitrary units (a.u.), to which the amplitudes of all other bursts within a recording period were normalized, according to previously described methods (Kienbaum *et al*. 2001 *b*; Fairfax *et al*. 2013). Identified bursts were inspected and scored (burst or no burst) by the operator. The MSNA was quantified as burst incidence (number of bursts per 100 heart beats), burst frequency (in bursts per minute), burst amplitude (i.e. strength) and total activity (product of burst frequency and mean burst amplitude). The location of each burst within the respiratory cycle was determined, and burst incidence, burst frequency, burst amplitude and total activity were calculated for each 10% time interval of the breath from the peak of inspiration (i.e. peak inspiration = time point 0).

**Figure 1:**
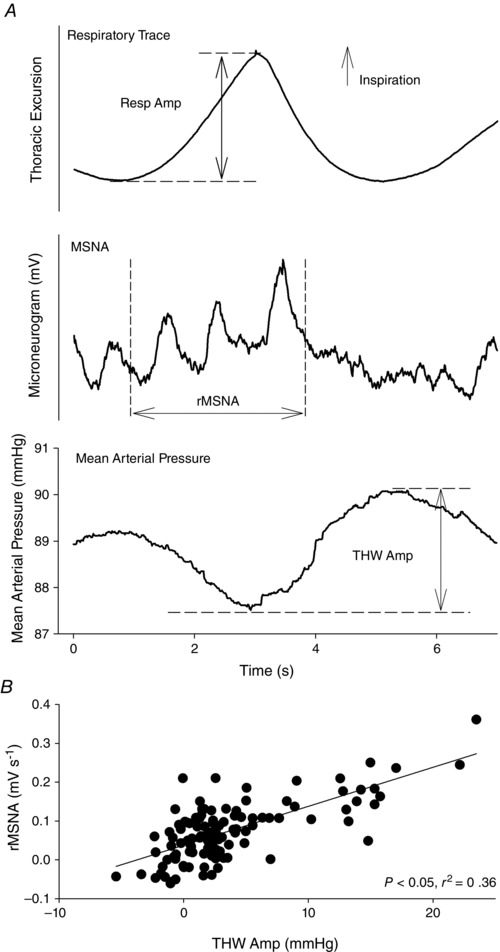
**Respiratory‐triggered waveform averaging of the raw respiratory, blood pressure and muscle sympathetic nerve activity (MSNA) signals (*A*) and sample respiratory‐mediated changes in MSNA (rMSNA) *versus* Traube–Hering wave amplitude (THW Amp) relationships (*B*) in one older subject** Following respiratory‐triggered averaging, regions of interest were set around the THW nadir, around the THW peak and around the MSNA associated with respiration (rMSNA). The rMSNA was quantified as the area under the sympathetic activity curve during the inspiratory period (i.e. from the beginning of the upslope to the peak of the thoracic excursion waveform) plus 50% of this duration, which thus extended into the postinspiratory period (*A*). For each subject, this approach was used to generate a breath‐by‐breath respiratory amplitude, THW Amp and rMSNA time series, and the association between these variables was examined (*B*). As the aim of this analysis was to assess the association between sympathetic neural events and THWs, no time shifting of the sympathetic neurogram was undertaken. Note that with a shift to account for conduction delays in MSNA (1.38 s in this subject), a sympathetic inhibition is apparent from approximately peak inspiration to end inspiration.

A novel time‐domain analysis was employed to examine the relationship between respiratory‐mediated changes in MSNA (rMSNA) and THW amplitude using a custom‐written interactive analysis program (Spike2; Fig. 1 *A*). Respiratory‐triggered averaging of the sympathetic neurogram and beat‐to‐beat MAP time series (obtained by the beat‐to‐beat integration of the arterial BP waveform over a cardiac cycle) was undertaken to identify temporal relationships between the THW amplitude and rMSNA. Regions of interest were set around the THW nadir, around the THW peak and around the MSNA associated with respiration (rMSNA). The rMSNA was quantified as the area under the sympathetic activity curve during the inspiratory period plus 50% of this duration, which thus extended into the postinspiratory period (Fig. 1 *A*). For each subject, this approach was used to generate a breath‐by‐breath respiratory amplitude, THW amplitude and rMSNA time series, allowing the relationships between these variables to be examined. For this analysis of the association between neural events and the THWs, sympathetic neurograms were neither time shifted nor normalized. This is in line with previous studies that have examined the associations between human sympathetic nerve activity and the ensuing vascular or blood pressure response (Fairfax *et al*. 2013).

Spontaneous cardiac baroreflex sensitivity was calculated using the sequence technique (Parati *et al*. 1995). A customized algorithm (Spike2) analysed the beat‐to‐beat time series of systolic BP, HR and R–R interval to identify sequences of three or more consecutive beats where systolic BP and HR changed in the opposite direction, or systolic BP and RR interval changed in the same direction. Once identified, a linear regression was applied to each sequence, and only sequences with an *r* ^2^ > 0.85 were accepted. Indices of cardiac baroreflex sensitivity were provided by the slope of the systolic BP *versus* HR and systolic BP *versus* R–R interval relationships, which have previously been shown to be correlated with traditional measures of resting operating point of the full cardiac baroreflex function curve (Robbe *et al*. 1987; Parlow *et al*. 1995).

To investigate the influence of age on arterial baroreflex control of MSNA, the relationships between diastolic BP *versus* burst incidence and total MSNA were examined, as previously described (Sundlöf & Wallin, 1978; Kienbaum *et al*. 2001 *b*). Briefly, diastolic BP for each cardiac cycle during the 10 min collection was grouped into 3 mmHg bins. For each diastolic BP bin, the percentage of cardiac cycles in which a burst occurred (burst incidence) and total burst amplitude divided by the number of cardiac cycles (total MSNA; expressed as arbitrary units per beat) were determined. Weighted linear regression analysis was used to determine the slope of the relationship between diastolic BP *versus* burst incidence and total MSNA. The mean *r* value of the slopes for young subjects was 0.90 ± 0.07 (range, 0.66–0.98) and for older subjects 0.89 ± 0.10 (range, 0.60–0.98).

Time‐ and frequency‐domain analyses were used to assess HR variability using commercially available software (Kubios HRV Software; Biomedical Signal Analysis Group, University of Kuopio, Finland) according to established methods (Task Force of the European Society of Cardiology and the North American Society of Pacing and Electrophysiology, 1996). The time‐domain methods for the assessment of HR variability included the square root of the mean of the sum of successive differences in R–R interval (RMSSD), standard deviation of all normal sinus R–R intervals (SDNN) and proportion of successive R–R intervals that vary by >50 ms (pNN50%). Spectral analysis of HR variability was undertaken using fast Fourier transformation and power spectral density determined at a high‐frequency range (HF, 0.15–0.4 Hz), a low‐frequency range (LF, 0.04–0.15 Hz) and between 0.0 and 0.4 Hz (total power, TP). The primary HR variability indices of interest are RMSSD, SDNN, pNN50% and HF, which have been generally indicated as respiratory sinus arrhythmia‐associated indices of cardiac parasympathetic activity (Task Force of the European Society of Cardiology and the North American Society of Pacing and Electrophysiology, 1996); however, interpretation of the physiological correlates for such HR variability parameters is controversial (Eckberg, 1997).

### Statistical analysis

Statistical analysis was performed using Statistical Package for the Social Sciences (SPSS) software, version 19.0 for Windows (SPSS Inc., Chicago, IL, USA). Baseline subject characteristics were compared using Student's unpaired *t* test. Mixed between‐ and within‐subjects ANOVA, adjusted using the Greenhouse–Geiser correction, was used to examine the main effects of respiratory phase, age group and their interaction. Spearman correlation analysis was used to evaluate relationships between breath‐by‐breath values of respiratory waveform amplitude (e.g. index of breath depth), rMSNA and THW amplitude (Fig. 1). A χ^2^ analysis was employed for comparisons of categorical data. Data are expressed as means ± SD, except where specified. A *P* value of <0.05 was considered statistically significant.

## Results

### Subject characteristics

**Figure 2:**
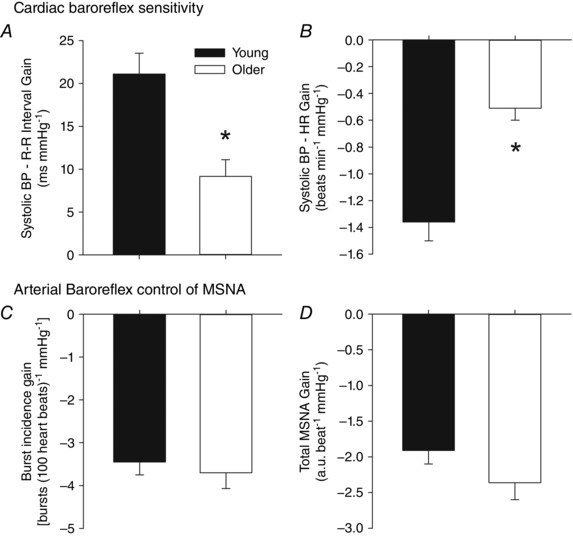
**Spontaneous measures of cardiac baroreflex sensitivity (*A* and *B*) and arterial baroreflex control of MSNA (*C* and *D*) in young and older groups** *P < 0.05 *versus* young. Cardiac baroreflex sensitivity was significantly lower in the older group compared with the young group, whereas no age‐related differences in arterial baroreflex control of MSNA were noted.

The mean age difference between young and older groups was 36 years (Table 1). Young and older participants were matched for body mass index (25 ± 3.5 *versus* 26 ± 4.0 kg m^−2^, respectively, *P = *0.82). Mean arterial blood pressure was significantly higher in the older group (95 ± 8.9 mmHg) than in the young group (86 ± 8.2 mmHg, *P = *0.04), whereas HR (60 ± 7.9 beats min^−1^ in the young group *versus* 62 ± 17.2 beats min^−1^ in the older group, *P = *0.81) and respiratory rate (15.3 ± 1.8 breaths min^−1^ in the young group *versus* 13.4 ± 2.4 breaths min^−1^ in the older group, *P = *0.06) were similar. The THW amplitude was also similar between groups (2.0 ± 0.9 mmHg in the young group *versus* 2.7 ± 1.3 mmHg in the older group, *P = *0.19), but as anticipated MSNA burst incidence [22.7 ± 9.2 *versus* 42.2 ± 13.7 bursts (100 heart beats)^−1^, *P = *0.002] and burst frequency (13.5 ± 6.0 *versus* 25.0 ± 7.6 bursts min^−1^, *P = *0.001) were higher in the older group. Spontaneous cardiac baroreflex sensitivity was lower in the older group compared with the younger group (*P* < 0.001), while arterial baroreflex control of MSNA was similar between groups (Fig. 2).

**Table 1:** **Subject characteristics**

Characteristic	Young group	Older group	*P* Value*
Age (years)	22 (2)	58 (6)	<0.001
Weight (kg)	79 (11)	81 (18)	0.75
Height (cm)	177 (5)	177 (9)	0.97
SBP (mmHg)	123 (14.0)	136 (14.3)	0.046
DBP (mmHg)	68 (5.6)	74 (6.3)	0.04

Values are shown as the means (SD). Abbreviations: DBP, diastolic blood pressure; and SBP, systolic blood pressure. *P < 0.05, Student's unpaired *t* test.

### Respiratory–sympathetic coupling

**Figure 3:**
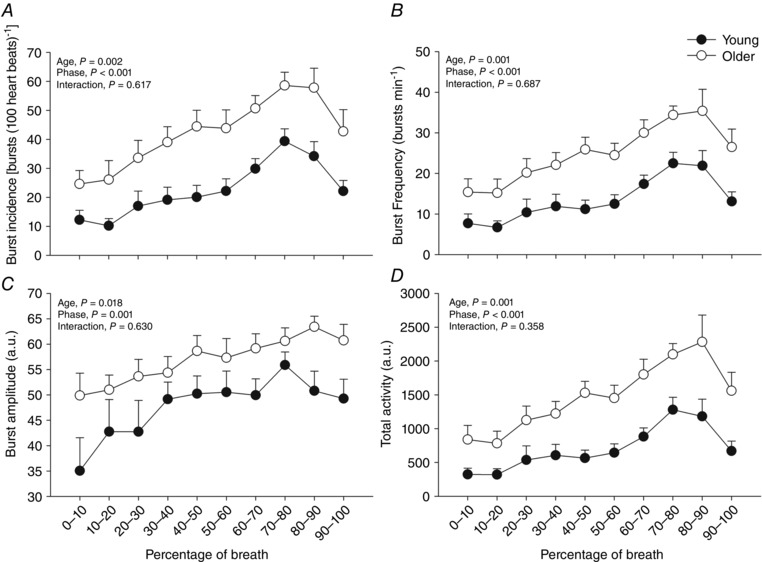
**Respiratory modulation of MSNA burst incidence (*A*), frequency (*B*), amplitude (*C*) and total activity (*D*) in young and older groups** Peak of inspiration is at zero. Despite MSNA being elevated in the older group, no age‐related alterations in the effect of respiratory phase on MSNA were noted (i.e. no significant interaction was observed between age and respiratory phase). Thus, the normal inspiratory‐linked inhibition of MSNA was preserved in the older subjects.

**Figure 4:**
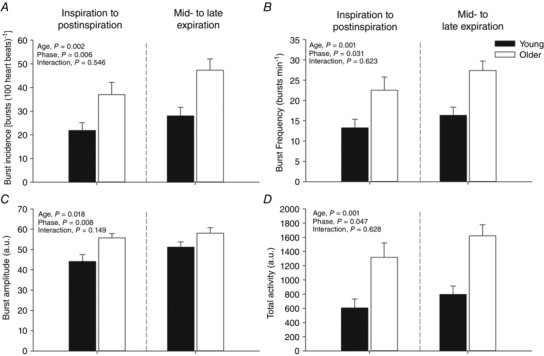
**Respiratory modulation of MSNA burst incidence (*A*), frequency (*B*), amplitude (*C*) and total activity (*D*) during the inspiratory to postinspiratory period and mid‐ to late expiration in young and older groups** Note that sympathetic neurograms were shifted in time to account for conduction delays calculated according to subject height. Mid‐ to late expiration was taken as 30–80% of the breath and the inspiratory to postinspiratory period taken as 0–30 and 80–100% of breath as presented in Fig. 3.

The influence of respiration on MSNA parameters in young and older participants is summarized in Figs 3 and 4. All parameters of MSNA examined (burst incidence, burst frequency, burst amplitude and total activity) were significantly higher in the older group in all respiratory phases (*P* < 0.05, ANOVA main effect of age). The MSNA was modulated by respiration, such that MSNA burst incidence, burst frequency, burst amplitude and total activity were lowest around the mid‐inspiratory to postinspiratory period and highest during mid‐ to late expiration (*P* < 0.05, ANOVA main effect of respiratory phase). *Post hoc* analysis showed that compared with 0–10% of the breath (0% = peak of inspiration), MSNA was significantly higher for burst incidence at 40–50 and 60–100% of the breath, higher for burst frequency and total activity at 60–100% of the breath, and higher at 70–80% of the breath for burst amplitude (Fig. 3). Importantly, the magnitude of the respiratory modulation of all parameters of MSNA was similar in both groups [i.e. no significant statistical interactions were observed between age group and respiratory phase (ANOVA)]. More specifically, MSNA burst incidence, burst frequency, burst amplitude and total activity were significantly higher in the older group compared with the younger group during both the mid‐ to late expiratory period and the inspiratory to postinspiratory period (Fig. 4). However, a similar degree of inspiratory attenuation of MSNA was observed in both groups (i.e. a significant main effect of respiratory phase was observed, but a significant interaction between age and phase was not), such that MSNA was significantly lower during the inspiratory to postinspiratory period compared with the mid‐ to late expiratory period (Fig. 4). The RMSSD, SDNN, pNN50%, HF, LF and TP were significantly lower in older group compared with the younger group, while LF/HF was higher in the older group (Table 2).

**Table 2:** **Time‐ and frequency‐domain measures of heart rate variability in young and older subjects**

Parameter	Young group	Older group	*P* Value
RMSSD (ms)	49.3 (37.2–74.4)	20.2 (16.8–36.3)	0.004
SDNN (ms)	64.2 (18.7)	42.6 (16.1)	0.013
pNN50 (%)	29.2 (16.7–48.8)	2.2 (1.4–10.9)	0.003
HF (ms^2^)	981 (633–1593)	152 (112–234)	0.002
LF (ms^2^)	869 (475–1817)	407 (211–681)	0.02
TP (ms^2^)	3288 (2624–5848)	1218 (857–3381)	0.02
HF (n.u.)	50.1 (12.4)	34.7 (19.5)	0.05
LF (n.u.)	49.9 (12.4)	65.3 (19.5)	0.05
LF/HF	1.13 (0.59–1.43)	2.17 (1.54–3.84)	0.02

Values are shown as the means (SD) or means (interquartile range). Abbreviations: HF, high‐frequency power (0.15–0.4 Hz); LF, low‐frequency power (0.04–0.15 Hz); n.u., normalized units; pNN50%, proportion of successive R–R intervals that vary by >50 ms; RMSSD, square root of the mean of the sum of successive differences in R–R interval; and SDNN, standard deviation of all normal sinus R–R intervals. *P < 0.05, Student's unpaired *t* test or Wilcoxon signed rank test.

### Respiratory‐related MSNA–BP coupling

The proportion of participants in whom there were significant correlations between parameters of rMSNA–BP coupling [THW amplitude, rMSNA, respiratory waveform amplitude (i.e. breath depth)] is summarized in Table 3. The rMSNA positively and significantly predicted the magnitude of the following THW (lag 0) in 100% of young (Fig. 1 *B*) and 80% of older subjects (*P = *0.136 young *versus* older group). This positive correlation was not observed with the magnitude of the THW amplitude associated with the next breath (lag +1); indeed, this showed a negative correlation in 80% of young and 70% of older subjects (*P = *0.606 young *versus* older groups). In some individuals (30% of young and 50% of older subjects, *P = *0.361), a significant correlation was noted between THW amplitude and the following rMSNA; however, in the majority, this relationship was positive (counter to the expected relationship). This test of reverse causation (i.e. whether the rMSNA related to the magnitude of the preceding THW through the engagement of the arterial baroreflex) was thus not proved and was less common than the proportion of individuals in whom a correlation between rMSNA and the following THW was observed. In 60% of the young group and 50% of the older group, the respiration waveform amplitude was significantly and positively correlated with the following THW amplitude, which was not different between groups (*P = *0.653 young *versus* older groups).

**Table 3:** **Proportion of participants in whom significant correlations between parameters of respiratory‐related MSNA–BP coupling were observed**

Correlation	Young group (*n* = 10)	Older group (*n* = 10)	*P* Value
Respiratory Amp *versus* THW Amp, direction of correlation (*r*)	60% positive [0.28 (0.07)]	50% positive [0.22 (0.05)]	0.65
rMSNA *versus* THW Amp, direction of correlation	100% positive [0.45 (0.07)]	80% positive [0.48 (0.19)]	0.14
rMSNA *versus* THW Amp (lag +1), direction of correlation (*r*)	80% negative [−0.31 (0.09)]	70% negative [−0.34 (0.09)]	0.61
Previous THW Amp *versus* rMSNA, directions of correlation (*r*)	30%, one negative [−0.19], two positive [0.20 (0.02)]	50%, one negative [−0.18], four positive [0.20 (0.04)]	0.36
Respiratory Amp *versus* rMSNA, directions of correlation (*r*)	50% positive [0.34 (0.11)]	30%, two negative [−0.24 (0.04)], one positive (0.60)	0.36

Abbreviations: *r*, correlation coefficient; Respiratory Amp, amplitude of the respiratory waveform excursion (e.g. index of breath depth); rMSNA, respiratory‐linked muscle sympathetic nerve activity; and THW Amp, amplitude of the Traube–Hering wave. Data are shown as means (SD).

## Discussion

The major novel findings of our investigation are twofold. First, in contrast to our initial hypothesis, the strength of the respiratory modulation of the MSNA parameters (e.g. burst incidence, frequency, amplitude and total activity) was preserved in healthy older individuals. Second, we identified a significant association between the respiratory‐mediated changes in MSNA (rMSNA) and THW amplitude, and that this was similar in healthy young and older groups. Collectively, these findings suggest that an attenuation of inspiratory‐linked inhibition of MSNA does not appear to explain the elevated resting MSNA in older individuals, and that central respiratory–sympathetic coupling is a component of the THWs in both young and older humans.

A respiratory modulation of sympathetic nerve activity is evident in recordings from rats (Simms *et al*. 2009), cats (Boczek‐Funcke *et al*. 1992) and humans (Eckberg *et al*. 1985, 1988; Seals *et al*. 1990; Seals, 1993; St Croix *et al*. 1999; Dempsey *et al*. 2002), with the exact pattern of respiratory modulation of SNA being species and target organ specific. In adult rats, muscle vasoconstrictor‐type sympathetic neurones are typically inhibited during early inspiration, with a peak of activity observed during the mid‐inspiratory to postinspiratory phase (i.e. first part of expiratory interval) and sometimes a smaller peak in late expiration (Simms *et al*. 2009). Importantly, this pattern of respiratory–sympathetic coupling is altered in several rat models of hypertension, such that sympathetic activity becomes particularly enhanced during inspiration and appears to be a causative factor in the increased vascular resistance, BP and potentially end‐organ damage in these animals (Zoccal *et al*. 2008; Simms *et al*. 2009; Toney *et al*. 2010; Zoccal & Machado, 2010). Increased sympathetic activity in patients with chronic heart failure has also been linked to alterations in respiratory–sympathetic coupling such that the MSNA is highest in those patients in whom the normal inspiratory‐linked inhibition of MSNA is most diminished (Goso *et al*. 2001). An attenuation of the direct inhibitory effect of pulmonary stretch receptors on sympathetic activity in response to lung inflation could potentially explain this observation (Gerber & Polosa, 1978; Yu *et al*. 1990), although an enhanced central respiratory coupling that elevates MSNA during inspiration remains a possibility. Given the well‐established increase in sympathetic nerve activity in older individuals (Seals & Esler, 2000), we expected to observe a reduced inspiratory inhibition of MSNA in older compared with young participants. However, a clear inspiratory attenuation of MSNA was evident, and no statistical interaction between age and respiratory cycle phase was observed. This indicates that a diminished inspiratory‐linked inhibition of MSNA in the older group does not contribute to the elevated MSNA in the older individuals. Nevertheless, it is important to note that all indices of MSNA studied were significantly higher in the older group compared with the younger group at all respiratory phases examined, which may represent an enhancement of respiratory drive to MSNA across the entire respiratory cycle, although the temporal pattern of the modulation is similar. Given the similar arterial baroreflex regulation of MSNA in young and older individuals observed in the present study and reported by others (Ebert *et al*. 1992), this also appears to be an unlikely explanation. However, impaired cardiopulmonary baroreflex buffering of MSNA (Cleroux *et al*. 1989), elevated brain noradrenaline activity (Esler *et al*. 2002) and/or enhanced peripheral afferent drive from, for example, the heart (Malliani & Montano, 2002), kidney (Johns & Abdulla, 2013) or carotid body (McBryde *et al*. 2013) remain as potential mechanisms for the elevated MSNA observed in the older group.

Using cross‐correlation histograms between the sympathetic spikes and respiratory‐related chest excursion signals, Fatouleh & Macefield (2011) reported a similar respiratory modulation of MSNA in groups of young (29 ± 2 years old) and middle‐aged individuals (∼20 years older), although no respiratory‐mediated fluctuations in BP were detected. The findings of the present study partly support these observations and extend them by determining whether sympathetic burst occurrence (i.e. incidence) and strength (i.e. amplitude) are differentially modulated within a breath. Both animal (Malpas & Ninomiya, 1992; Malpas *et al*. 1996) and human investigations (Kienbaum *et al*. 2001 *b*) have identified that the arterial baroreflex differentially modulates sympathetic burst incidence and amplitude. Previous work examining respiratory modulation of MSNA bursts in humans has focused on the evaluation of MSNA in terms of total activity (Eckberg *et al*. 1985, 1988; Seals *et al*. 1990, 1993; St Croix *et al*. 1999; Dempsey *et al*. 2002). We observed that all indices of MSNA parameters examined (e.g. burst incidence, burst frequency, amplitude and total activity) were significantly modulated by respiration and, despite an age‐related elevation in all parameters, no significant interactions between age and respiratory phase were observed.

Our study uses a novel methodological approach to examine the relationships between respiratory‐mediated changes in MSNA and arterial BP (Towie *et al*. 2012). Whilst animal experiments support the contention that respiratory modulation of vasomotor sympathetic outflow causes phasic changes in arteriolar smooth muscle tone, thus generating THWs (Simms *et al*. 2009), the results of human work is more equivocal (Conci *et al*. 2001; Zhang *et al*. 2002). In several recent studies, the ability of spontaneously occurring MSNA bursts to evoke a beat‐to‐beat change in peripheral vascular resistance and blood pressure has been carefully described (Vianna *et al*. 2012; Fairfax *et al*. 2013). In accordance with these reports, we observed significant association between the respiratory‐mediated changes in MSNA (rMSNA) and the THW amplitude of the following breath in all of the young individuals and the large majority of older individuals studied. This relationship was evident in a similar proportion of young and older individuals. This was somewhat surprising given the reported age‐related reduction in α‐adrenergic responsiveness (Esler *et al*. 1995; Dinenno *et al*. 2005) and may be related to the reported downregulation of uptake mechanisms and/or downregulation of degrading enzymes for noradrenaline (Esler *et al*. 1995), or indeed, it may be the case that despite a reduction in α‐adrenergic responsiveness there is a sufficient safety margin for transmission at the neurovascular junction to maintain effective sympathetic signalling. Notably, the respiratory‐mediated changes in MSNA were not robustly associated with the previous THW amplitude, supporting the contention that respiratory modulation of MSNA is independent of fluctuations in BP (Seals *et al*. 1990, 1993).

Our data support the contention that respiratory modulation of vasomotor sympathetic outflow causes phasic changes in arteriolar smooth muscle tone, thus generating THWs. However, it is important to appreciate that a number of complex feedforward and feedback mechanisms have also been implicated (Zhang *et al*. 2002; Tan & Taylor, 2010). Indeed, Tan & Taylor (2010) demonstrated that respiratory fluctuations in heart period cause arterial BP fluctuations, especially in young healthy individuals, rather than buffering such pressure fluctuations. In the present investigation, a clear reduction in the respiratory‐linked fluctuations of heart period was noted in the older group; however, as in a previous investigation (Fluckiger *et al*. 1999), respiratory‐linked fluctuations in BP were not significantly different in the young and older groups. Nevertheless, further studies are required to determine how age changes the relative contribution of the many mechanisms implicated in the generation of THWs.

In the present study, MSNA was examined because of its well‐established importance in BP regulation. The inability to record directly from the sympathetic nerves supplying the renal or splanchnic vascular beds in humans means that the potential contribution from these regions to THW amplitude was not ascertained. Furthermore, a definitive explanation for the age‐related elevation in BP remains elusive. Aside from adrenergic mechanisms, a number of other factors remain as possible contributors, including increased arterial stiffness (Avolio *et al*. 1985), upregulation of endothelin‐1‐mediated vasoconstriction (Van Guilder *et al*. 2007), reductions in endothelial nitric oxide bioavailability (Eskurza *et al*. 2004) and alterations in renin–angiotensin–aldosterone pathways (Tsunoda *et al*. 1986). Of note, the higher BP with a concomitant preservation in the sensitivity of arterial baroreflex control of MSNA observed in the older group may be explained by the resetting (or shift of the set‐point) around which MSNA is regulated. However, a limitation of the method employed to assess arterial baroreflex function in the present study was that a complete assessment of the full stimulus–response relationship is not provided, and for this a more direct method is required (e.g. modified Oxford technique). Furthermore, while the ‘spontaneous’ index of arterial baroreflex function we have used has been considered to provide a useful indicator of sensitivity around the prevailing BP (i.e. operating point of the full baroreflex function curve; Young *et al*. 2010), it is poorly associated with sensitivity measures derived using the modified Oxford technique (Hart *et al*. 2010).

As in many human studies examining respiratory modulation of sympathetic nerve activity, we assessed respiration using a strain‐gauge pneumobelt (Han *et al*. 1997; Peng *et al*. 2002). The advantage of this approach is that it is unobtrusive and avoids participants having to breathe through a mouthpiece, which almost inevitably tends to alter the breathing pattern (Han *et al*. 1997; Peng *et al*. 2002). The potential disadvantage of this approach is that the time delay between the occurrence of respiratory‐related events within the central nervous system and changes in thoracic circumference (as well as delays between respiratory central pattern generators and MSNA) is not accounted for, and we have assumed that this is a constant between young and older groups. It should also be noted that as in several other cross‐sectional studies of respiratory sympathetic coupling (Fatouleh & Macefield, 2011, 2013) we did not control respiratory rate and depth. However, importantly, it has been reported that MSNA is no different during uncontrolled spontaneous breathing and controlled breathing at 12 breaths min^−1^ (Eckberg *et al*. 1985). Another consideration is that with human sympathetic nerve recordings we do not know the exact location/proximity of the recording electrode tip to the sympathetic fascicles in the nerve, and therefore, burst amplitude has to be expressed in normalized units. Although this may restrict the fidelity with which we can make between‐group assessments of cyclical fluctuations in sympathetic activity, there is currently no way around this with direct human sympathetic nerve measures. As such, care should be taken when making comparisons with animal investigations where this is less of a consideration (Simms *et al*. 2009, 2010).

In conclusion, our data suggest that despite an age‐related elevation in MSNA the strength of the respiratory modulation of MSNA is similar in young and older individuals. Indeed, the normal inspiratory‐linked inhibition of MSNA is preserved in older individuals. This suggests that the elevation in MSNA found in older individuals is unrelated to a diminished respiratory–sympathetic coupling. In addition, respiratory‐mediated changes in MSNA appear to be a significant component of THW amplitude in both young and older groups.

## Additional information

### Competing interests

None declared.

### Author contributions

A.S. contributed to data analysis, data interpretation and drafting the manuscript. D.B.M. contributed to data analysis and critical review of the manuscript. G.Y.H.L. contributed to the study design and critical review of the manuscript. P.J.F. contributed to the data acquisition, data interpretation and critical review of the manuscript. J.F.R.P. contributed to study design, data interpretation and critical review of the manuscript. A.E.P. contributed to study design, data analysis, data interpretation and critical review of the manuscript. J.P.F. contributed to study design, data acquisition, data analysis, data interpretation and critical review of the manuscript. All authors approved the final version of the manuscript.

### Funding

These studies were funded in part by British Heart Foundation project grant PG/11/41/28893.
